# Genome-Wide Distribution of Nascent Transcripts in Sperm DNA, Products of a Late Wave of General Transcription

**DOI:** 10.3390/cells8101196

**Published:** 2019-10-03

**Authors:** Leila Kianmehr, Homayoun Khazali, Hassan Rajabi-Maham, Ali Sharifi-Zarchi, François Cuzin, Minoo Rassoulzadegan

**Affiliations:** 1 Animal Sciences and Biotechnology Department, Faculty of Life Sciences and Biotechnology, Shahid Beheshti University, G.C, Tehran 1983963113, Iran; L_kianmehr@sbu.ac.ir (L.K.); homkhaz2@gmail.com (H.K.); H_Rajabi@sbu.ac.ir (H.R.-M.); 2 Université de Nice-Sophia Antipolis, Faculté des Sciences, Parc Valrose, INSERM-CNRS, 06108 Nice CEDEX 2, France; Francois.CUZIN@univ-cotedazur.fr; 3 Computer Engineering Department, Sharif University of Technology, Tehran 1458889694, Iran; asharifiz@gmail.com

**Keywords:** RNA, DNA–RNA hybrids, spermatozoa, transcripts

## Abstract

Mature spermatozoa contain a whole repertoire of the various classes of cellular RNAs, both coding and non-coding. It was hypothesized that after fertilization they might impact development, a claim supported by experimental evidence in various systems. Despite the current increasing interest in the transgenerational maintenance of epigenetic traits and their possible determination by RNAs, little remains known about conservation in sperm and across generations and the specificities and mechanisms involved in transgenerational maintenance. We identified two distinct fractions of RNAs in mature mouse sperm, one readily extracted in the aqueous phase of the classical TRIzol procedure and a distinct fraction hybridized with homologous DNA in DNA-RNA complexes recovered from the interface, purified after DNase hydrolysis and analyzed by RNA-seq methodology. This DNA-associated RNA (D RNA) was found to represent as much as half of the cell contents in differentiated sperm, in which a major part of the cytoplasmic material has been discarded. Stable complexes were purified free of proteins and identified as hybrids (R-loops) on the basis of their sensitivity to RNase H hydrolysis. Further analysis by RNA-seq identified transcripts from all the coding and non-coding regions of the genome, thus revealing an extensive wave of transcription, prior to or concomitant with the terminal compaction of the chromatin.

## 1. Introduction

The presence of significant amounts of RNA in the transcriptionally inert spermatozoon has been observed [1,2]. On the other hand, microinjection in fertilized oocytes demonstrated RNA-mediated inheritance of epigenetic traits, showing that sperm RNAs act as transgenerational epigenetic determinants [3,4,5]. In addition, a complex set of sncRNAs potentially available for delivery upon fertilization [6,7] was revealed. After the elimination of the bulk of cytoplasmic RNA, the spermatozoon still contained, together with highly compacted DNA, a multiplicity of nuclear RNAs. A previous study revealed a population of chromatin-associated RNA in sperm [8], but nothing is known about the possible presence of DNA–RNA hybrids (R-loops). We found that a significant fraction of the RNA is in fact present as R-loop-like complexes, three-stranded structures composed of a RNA–DNA hybrid and a displaced single-stranded DNA strand [9]. To investigate their occurrence in sperm, we separately purified the free RNA from the water phase of TRIzol extracts and the DNA-bound molecules whose possible functions are thus open to analysis. We first performed a bioinformatics analysis of the profiles of DNA-associated and free RNA molecules. Non-coding RNAs such as long non-coding RNA (lncRNA) and small non-coding RNA including piRNA and tRNA-derived fragments also have been identified. The surprising result was that the distribution of these transcripts reflects the totality of the genome, including both coding and non-coding sequences. Assuming that their extent provides a picture of the ongoing transcription activity at the time of the final compaction of the chromatin, we conclude that a generalized wave of transcription is initiated at the final stage of differentiation, generating a peculiar “multiloop” structure of the incoming paternal genome.

## 2. Materials and Methods

### 2.1. Mice

The experiments described herein were carried out in compliance with the relevant institutional and French animal welfare laws, guidelines and policies. Sperm biopsies of animals (C57BL/6 genetic background) were kindly provided. The experiments were approved by the French ethic committee (‘Comité Institutionnel d’Ethique Pour l’Animal de Laboratoire’; file number NCE/2012-54).

### 2.2. Sperm Preparation

**Figure 1:**
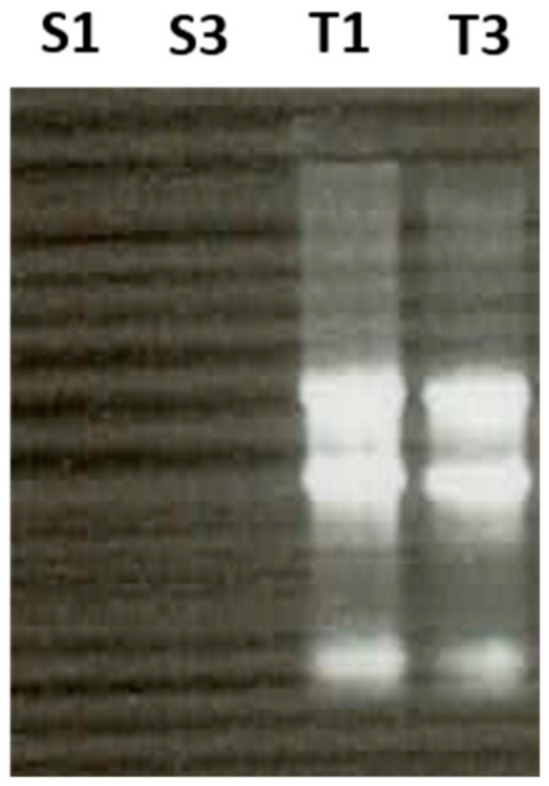
Absence of ribosomal RNAs (5, 18, 28 S) in sperm preparation (S1 and S3) compared to testis (T1 and T3). Total RNA extracted from purified sperm and from somatic testicular tissue was analyzed on ethidium bromide agarose gel electrophoresis (1 µg).

Mature mouse spermatozoa were isolated after swim-up from the cauda epididymides from individual mice (6 and 14 months old). These samples did not contain any somatic cells, as estimated by the most simple tests of spreading on slide, Giemsa staining and microscope checking. On the basis of this routine test, we can safely say that the level of contamination by somatic cells and germinal precursors is less than 1 cell in 10,000. Such a low estimate was confirmed by the absence of the 5, 18 and 28S ribosomal RNAs characteristic of mature sperm [3,6,10,11] (Figure 1). Motile spermatozoa were washed twice in MEM buffer (1 mM Na pyruvate, 0.5 mM EDTA, 50 U/mL penicillin, 50 mg/mL streptomycin and 0.1% BSA) by centrifugation. Sperm pellets were dissolved in high salt 5M Na Cl (hypertonic) submitted to osmotic shock by dilution in phosphate-buffered saline (PBS) and centrifuged again. The pellets of sperm cells were washed twice in 50 mM HEPES buffer pH 7.5, 10 mM NaCl, 5 mM Mg acetate and 25% glycerol.

### 2.3. Preparation of DNA-Bound RNA (D)

Extraction of total nucleic acids was performed by the separation of DNA and RNA with the standard TRIzolTM protocol [12]. TRIzol extracted interface materials were ethanol precipitated and followed by overnight incubation at 56 °C in Tris buffer 20 mM pH8, EDTA 50 mM, with 0.5% SDS, 20 µM dithiotheritol and 400 µg/mL proteinase K. After enzymatic removal of the proteins of total nucleic acids, samples were fractionated either through binding onto “ZymoSpinTM” columns according to the specifications of the manufacturer (ZYMO-RESEARCH CORP Irvine CA, USA) and elution columns or by chloroform extraction (www.zymoresearch.com). The free-RNA (R) fraction was separated and columns that retained the DNA fraction were treated first with RNase A and then with DNase. The RNA fraction that remained bound to the DNA was released and further purified. Gel electrophoresis and staining with ethidium bromide revealed a smear distribution from long to short oligonucleotides (not shown). RNase H treatment before Dnase treatment displaces the majority of the signals, while RNase A has no effect [13] (see Table S1).

### 2.4. RNA Sequencing (RNA-seq)

In total, 10 to 20 × 10^6^ spermatozoa were recovered from 2 adult males (6 and 14 months old). See above extraction of DNA-bound RNA. After DNase digestion, 10–100 ng of RNA was recovered and sent to Eurofins Genomics Eurofins Medigenomix GmbH, Ebersberg, Germany) for high-throughput sequencing on an Illumina HiSeq 2500 or Illumina MiSeq Genome Analyzer. RNA libraries representing biological replicates of sperm free-RNA and DNA-bound RNA fractions, each pooled from two males, consist of two technical replicates.

### 2.5. Library Characteristics

To characterize the transcripts in mouse sperm, we generated approximately 66 (D1, 6 months male), 69 (D3, 14 months male), 77 (R1, 6 months male) and 87 (R3, 14 months male) million reads mapped to unique sites in the wild-type mouse genome from D and R sperm samples. The average fragment length of the total reads was ~100 bp. More than 50% of reads mapped uniquely to the mouse genome. All the primary sequence characteristics for sample libraries of the D and R sequences of germ cells are summarized in the Supplementary Materials File S1 (Table S2). Sperm transcript profiling and alignment-based visualization were used for the performed analyses. Comparison of transcript abundance between samples was measured by RNA-seq yielded TPM (transcript per million) between different samples.

### 2.6. Transcript Distribution

RNA-seq analysis covers the whole genome with both fractions; however, the distribution of the transcripts was not completely identical to their identity and level. Variations were observed in all classes of RNAs from D to R fractions.

### 2.7. Bioinformatics Analysis

Quality control of the RAW sequencing reads was performed using FastQC 0.11.7. RNA-seq reads were processed by cutadapt v1.16 trimming adaptor sequences on the 3′ end with a 10% error rate. Based on the FastQC results, 10 nucleotides were cropped from the 5′ end of each read, and reads with a length of less than 20 bp were discarded. Samples were then directly quantified using Salmon v0.12.0 [14] on an index created from the GRCm38 transcript using GENCODE vM18, tRNA and piRNA precursors. Then, transcript counts were imported into tximport for all downstream analysis and were then summed by gene symbol [15]. Quantifications were also done using featureCounts. For differential transcript analysis, only protein-coding transcripts and long non-coding RNA that had at least 20 reads in each sample were fixed. Differential transcript analysis of the D and R fractions in mouse sperm RNA-seq samples was performed with DESeq2 (version 3.8) [16] and edgeR R package [17]. The *p*-values were adjusted using the Benjamini and Hochberg method [18]. An adjusted *p*-value (*P*adj) of 0.05 was set as the significant differential transcript threshold. Plots were generated using the ggplot2 R package (version 3.1.0) [19]. Hisat2 was used to align trimmed reads to the reference genome (mm10) [20]. we ensured quality of alignment using stats and plot-bamstats utilities of samtools [21]. Visualization of alignment was performed using Integrative Genome Viewer (IGV) version 2.5.2 [22]. Gene ontology (GO) enrichment and pathway analysis were carried out using the DAVID functional and enrichment tool [23]. The resulting GO terms with a corrected *p*-value of less than 0.05 were considered significantly enriched. For heatmaps, log2 (1-normalized counts) was used. RNA-seq reads were also quantified for tRNA-derived fragments and piRNA. An index of tRNA and piRNA sequences was constructed using tRNA sequences from GtRNAdb [24] and piRNAdb with parameters “--kmerLen 15′’. Percent identity matrix of the mapped reads was visualized using R package heatmap.

### 2.8. Quantitative RT-PCR Validation

Samples used for quantitative real-time PCR (RT-PCR) validation of the RNA-seq results were extracted as indicated above. The ten selected significant differential transcripts (Fezf2, Hmx3, Hoxb13, Nanog and Sox21; Lncenc1, Otx2os1, Platr30, Vmn1r51 and Uph) were confirmed by RT-PCR analysis. R and D extraction from sperm were implemented as indicated above. All primers (Table S3 in Supplementary Materials File S1) were designed. GAPDH was used as the housekeeping gene to normalize the qPCR transcript levels. RT-PCR was performed with a Light Cycler Instrument (Roche) using the miScript SYBR Green PCR Kit (Qiagen).

### 2.9. Statistical Analysis

Data are presented as means ± SEM. Group means comparisons are performed using Student’s *t*-test. Data are considered to be statistically significant with *p-*value < 0.05.

## 3. Results

### 3.1. Coding and lncRNA in Sperm Transcripts

**Figure 2:**
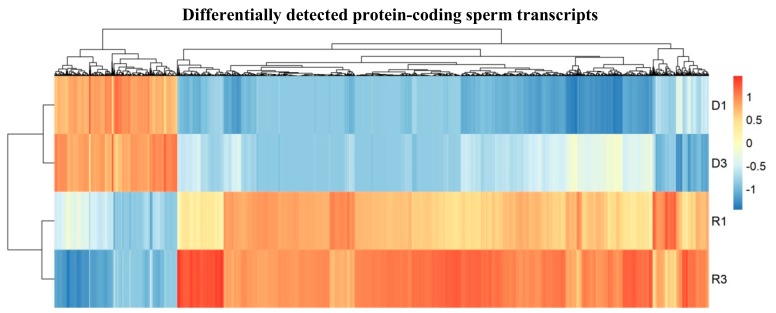
Heat map showing quantitative comparison of differentially detected protein-coding sperm transcripts from DNA-Bound RNA (D) to free-RNA (R) samples, which show that a lower range of significant specific transcripts were more present in D fraction samples (D1 and D3) than R fraction samples (R1 and R3); plotted are the row z-scores of log-normalized counts. (D n = 2 with two biological replicates in each sample group, R n = 2 with two biological replicates in each sample group, each replicate consists of sperm RNA pooled from two mice).

Assessment of the sperm transcripts by deep sequencing revealed that protein-coding transcripts are more abundant in R molecules in mature sperm compared to sperm D fractions. Hierarchical clustering was conducted for group transcripts with similar profiles between D and free fractions. To identify protein-coding and lncRNA transcripts potentially detectable in D versus R fractions, only transcripts that had at least 20 reads in all samples were tested. We compared the D versus R fractions for protein-coding and lncRNA transcripts, at first. Among them, some transcripts were significantly more present in D fraction samples (Log2FoldChang > 2, *P*adj 0 < 0.05) compared to the R fraction samples. Further quantitative comparison of protein-coding from D to R samples, however, showed the significant differential load of protein-coding transcripts. Comparison of D to R samples demonstrates that generally lower amounts of transcripts are found in D fractions (Figure 2 and Figure S1), which is expected because only one copy is hybridized in the natural DNA–RNA hybrids (R-loop).

**Figure 3:**
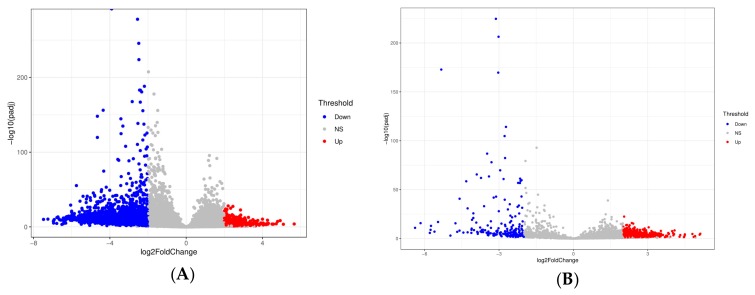
Volcano plot showing differentially detected transcripts for: (**A**) protein-coding and (**B**) lncRNA between the D and R fractions. Plots constructed using fold change (log2) and adjusted *p-*values to visualize the relationship between fold change and statistical significance. The x-axis corresponds to the fold change (log2) in transcript and the vertical axis represents the –log adjusted *p*-value. Significant differentially detected transcripts (*P*adj < 0.05) are indicated in red (higher level of transcripts in D vs. R; log2-fold change >2) and blue (lower level of transcripts in D vs. R; log2-fold change < −2).

In total, we identified 8317 differentially detected protein-coding transcripts (Supplementary Materials File S2). Among them, 1641 transcripts were found to be higher in the D fractions and were thus considered representative of the highly abundant sperm transcript class in the D fractions compared to the R fractions. The remaining 6676 transcripts showed lower level in free fractions (Figure 3A). Figure 3 represents the volcano plot that illustrates the statistical significance vs. log2-fold-change in differentially detected transcripts.

**Figure 4:**
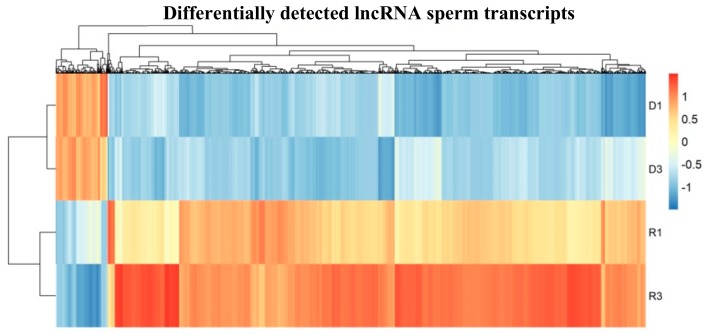
Heat map shows significant differential load of accumulated lncRNA in D sperm fractions in comparison to R fractions; (n = 2 with two biological replicates in each sample group). Plotted are the row z-score of log-normalized counts.

Significant differentially detected lncRNAs, either up or down, were found between the two groups through volcano plot (Figure 3B). For lncRNA hierarchical clustering demonstrated distinguishable lncRNA transcript profiling among samples (Figure 4). In further quantitative comparison of lncRNA transcript profiling data, we found 2175 differentially detected lncRNAs in mouse sperm using RNA-seq analysis. In total, 189 higher and 1986 lower levels of lncRNA transcripts were significant (log2-fold-change > 2, *P*adj < 0.05) between D and R sperm samples (Supplementary Materials File S3, Figure 4). See Figure S2 for single lncRNA transcripts in comparison with differential accumulation in sperm D fractions.

Comparison of D to R sperm samples showed more differences in terms of transcript features than genes (data not shown). Figure S3 demonstrates the heat map of pairwise Pearson correlation coefficients among all samples and shows that D samples are clustered together and D1 (sperm sample of 6-month-old mice) and D3 (sperm sample of 14-month-old mice) are similar, and different from the free fraction, while R (R1 and R3) samples are not clustered together, which may reflect the difference in age of the animal used (Figure S3).

Exon profiles of sperm samples were highlighted for free sperm RNA compared to DNA-associated RNAs. D and R sperm samples demonstrate more closed percent identity in exon features to protein-coding and lncRNA transcripts. On the other hand, D fraction shows a greater abundance of exon features than R fractions (Figure S4).

Several classes of small non-coding transcripts were also present within the DNA–RNA hybrid sperm libraries relative to R. Hierarchical clustering of differentially detected tRNA-derived fragments and piRNA in D compared to R revealed that R1 and R3 samples are not clustered together again for small non-coding transcripts, which may reflect the difference in age of the animal used (Figure S5).

### 3.2. Overview of Transcripts Analysis

**Figure 5:**
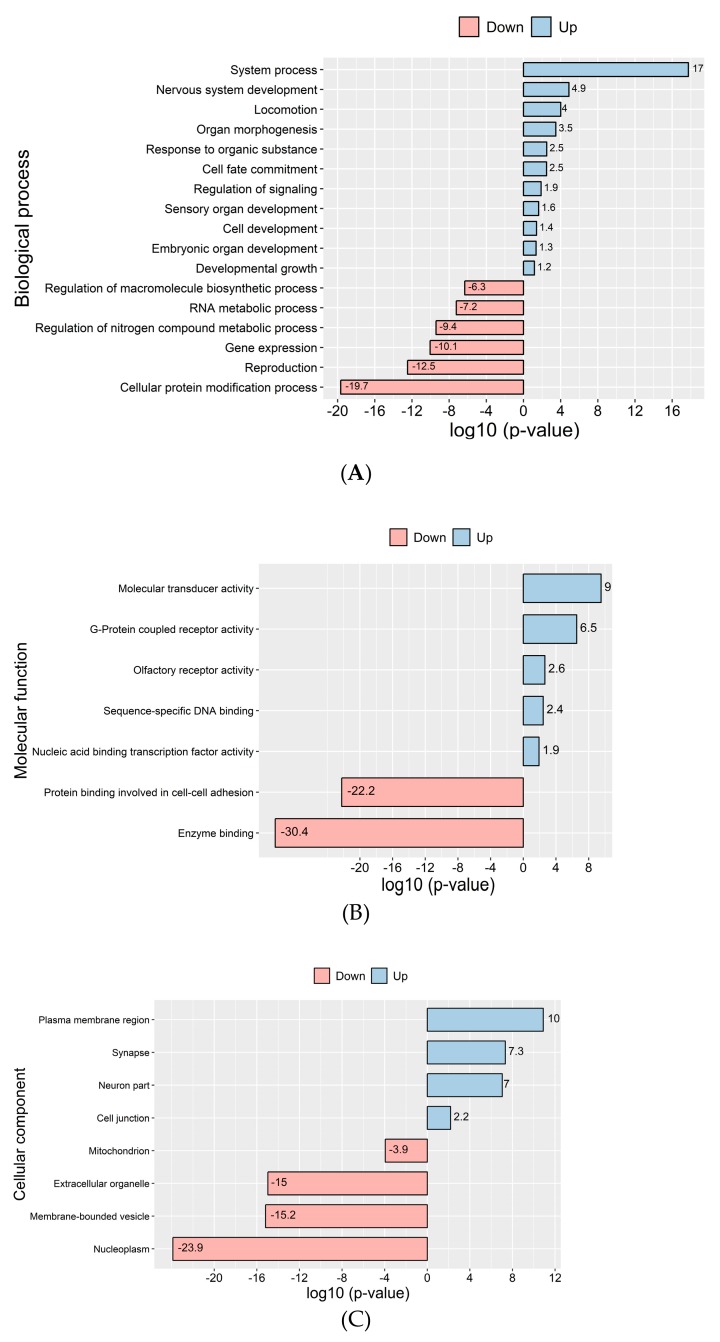
GO term analysis reveals enrichment of differentially detected protein-coding transcripts in sperm D and R fractions: (**A**) Bar graphs showing gene ontology analysis based on biological process, (**B**) cellular component (**C**) and molecular function. Red and blue bars represent –log10 (*p-*value) of each GO term enrichment. Up = higher level of transcripts in D vs. R, down = lower level of transcripts in D vs. R.

GO term enrichment analysis of reads mapping to differentially detected transcripts in D versus R molecules in sperm (log2-fold change >2, *P*adj < 0.05) were assessed with DAVID Bioinformatics Resources [25]. Figure 5 shows most significant GO terms were enriched for the biological process, cellular component and molecular function, among other things (see Figure 5A, Supplementary Materials File S5), and clearly reflect transcripts for critical functions. These results showed that most of the RNAs found in D fraction sperm samples versus R fraction were related to most transcription networks. The compartments to which the most significant transcripts were localized and molecular functions are demonstrated (Figure 5B,C and Supplementary Materials File S5). KEGG pathway analysis indicated that the transcripts in the D fractions participated in the olfactory transduction interaction, neuroactive ligand-receptor interaction and signaling pathways. However, the transcripts found in the R fractions were involved in the metabolic pathways, protein processing and ribosome (Supplementary Materials File S5). The GPCR, olfactory receptor, homeobox (conserved site) and homeodomain are the most significant protein domains in the D fractions (Supplementary Materials File S5). To identify the transcription factor target transcripts in D sperm molecules, we used Enrichr to investigate overrepresented transcription factor binding motifs in the regions of transcripts whose level was up twofold in D fractions vs. R fractions. Among 1721 transcription factors, 257 had the most significance (adjusted *p*-value <0.05) (Supplementary Materials File S4). Enrichr Submissions TF-Gene Co-Occurrence determined related transcription factors with the highest scores (Table S4).

### 3.3. Visualization

**Figure 6:**
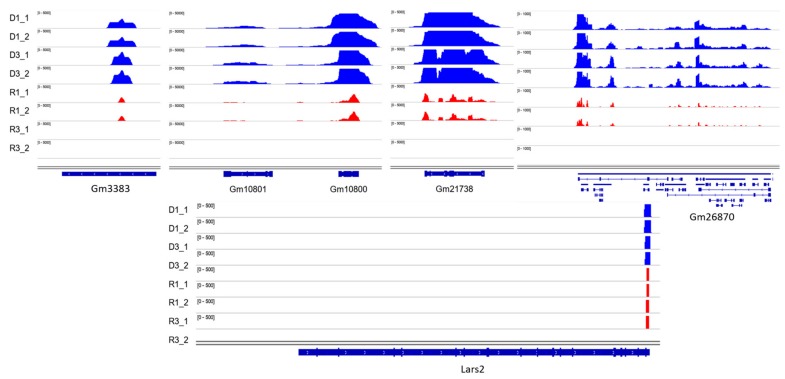
IGV visualization of a number of most significant differentially detected transcripts. The middle part of the figures illustrates the signals of the estimated fragment size in the top four tracks (blue) for D fractions vs. R fractions (red). The bottom section, labeled Refseq genes, demonstrates the gene annotation spans of the mouse genome annotation GRCm38. D = DNA-bound RNA; R = free RNA; (1 and 2 for each sample are technical replicates).

Hisat2 was used to align trimmed RNA-seq reads to the reference genome (mm10), then aligned reads to the whole genome are visualized in D samples versus R using IGV, the differences between R and D might reflect the better preservation of the transcripts in the D fraction (Figure 6). Among differentially detected lncRNA transcripts Gm3383 and Gm26870 as a long intergenic non-coding RNA (lincRNA) has very important difference significantly between D and R fractions. Among most significant differentially detected protein-coding transcripts Gm10800, Gm10801, Gm21738, Gm10721, Gm10720, Gm10717, Gm10719, Gm10722, Gm11168, Gm17535, Gm10715 and Lars2-209 are in high level in D fractions vs. free fractions (Figure 6). To confirm by depth-sequencing the lack of the 18S and 28S ribosomal RNAs in our sperm preparations, RNA-seq data of embryonic stem cells (Escs) wild-type as somatic cells was used [26]. Visualization of normalized reads showed us there are massive ribosomal sequences (18 and 28S precursor) in somatic cells versus sperm (Figure S6).

### 3.4. Quantitative Real-Time PCR Analysis

**Figure 7:**
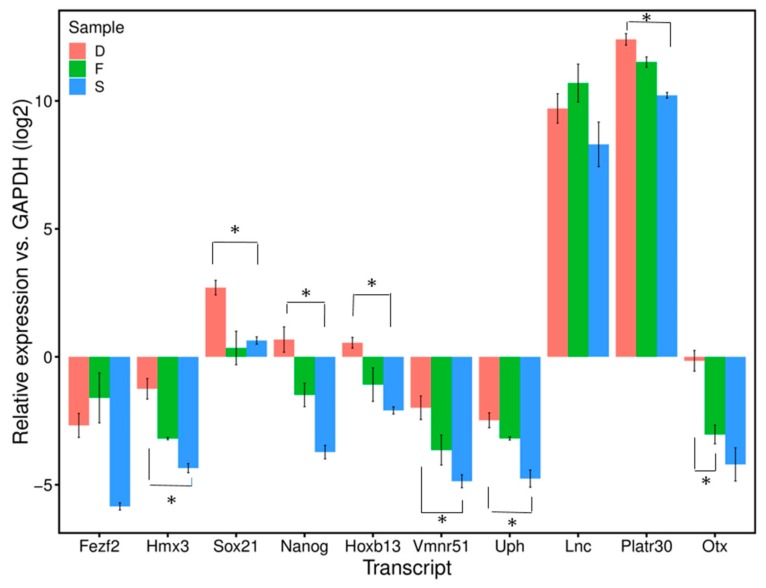
Relative expression level of ten candidate transcripts in sperm DNA-bound RNAs (D), free-RNAs (F) and somatic cells (S) vs. GAPDH (log2). The levels in each sample were normalized to that of GAPDH (log2) (internal control). Data are presented as means ± SEM. Group mean comparisons are performed using Student’s *t*-test.

RNA-seq analysis covers the whole genome, and assessment of the sperm RNAs by deep sequencing reflects the total transcripts. Among differentially detected protein-coding and lncRNA sperm RNAs, ten randomly selected transcripts which are in higher level (log2-fold-change >2) in D fractions, described to be involved in the development, were measured using quantitative real-time PCR. qPCR analysis (see below) confirmed the results of the RNA-seq. The relative transcripts of ten selected RNAs (Fezf2, Hmx3, Hoxb13, Nanog and Sox21) (Lncenc1, Otx2os1, Platr30, Vmn1r51 and Uph) in D fractions versus R were confirmed by quantitative real-time PCR (qPCR) using R and D extractions obtained from the sperm of wild-type mice. The transcript quantities were normalized to GAPDH as a reference gene (Figure 7). The transcript levels of 10 loci were higher in sperm D samples than R ones, which was consistent with the variations from RNA-seq analysis D fractions compared to R ones (*p* < 0.05). Furthermore, we also measured these transcripts in somatic cells, such as the brain and liver and a comparison of the means ± SEM of the D fraction in sperm to the D fraction in somatic cells showed a significant difference between most of them (*p* < 0.05); the level of transcripts was higher in sperm than somatic cells (Figure 7). Details of the known function of these short list transcripts are summarized in Table S5.

## 4. Discussion

Our initial motivation to study the sperm transcriptome was the discovery that RNAs transferred during fertilization may act in the control of gene expression and establishment of specific phenotypes in the progeny [3,4,5,27,28,29,30]. The experimental procedure relied on microinjection into mouse fertilized oocytes of a solution of sperm RNA prepared by conventional mean and corresponding to the R fraction of the present report. We now show that, in addition to these free RNAs, a significant fraction (DRNAs) was found bound to DNA and, as such, most likely transferred to the oocyte. Still, current evidence does not support the hypothesis that it might play a role in the formation and expression of the new genome. To the contrary, it is clear that any RNA hybridized on either strand of the DNA duplex would severely interfere with the progress of the replication fork [31] during the first cycle that takes place shortly after fertilization [32]. The various forms of genomic instability by “excessive R-loops” expected from studies in model systems would prevent any further development and, in fact, suppression of R-loops is insured by a protein machinery which eliminates the bound RNAs [33]. We therefore have to consider the bound D-RNAs as a feature of the spermatozoon not expected to play a role at any time post-fertilization. A possible function in the spermatozoon itself, for instance in the structure of the chromosomal DNA, while not documented at this stage may be considered for further studies. The most positive information at this stage is the view that the multiple RNA–DNA structures identified in sperm DNA are most likely to be remnants of transcription complexes arrested at the time of chromatin compaction. It is clear from the bioinformatics analysis that the totality of the chromosomal sequences, coding and non-coding, is represented in these RNAs. Our results reveal for the first time DNA–RNA hybrids in “frozen” transcriptional complexes in chromatin largely compacted by the replacement of histones by protamines.

RNA-seq analysis led us to conclude that the transcripts identified correspond to the entirety of the genome, including both coding and non-coding regions. The relative levels of a series of the abundant transcripts previously identified in spermatid RNAs (Fezf2, Hmx3, Hoxb13, Sox21, Otx2os1, Lncenc1, Platr30, Vmn1r51 and Uph) were assessed [34] all transcripts were found higher in the D molecules of sperm than in the R fraction and most of them are significantly higher than in those of somatic cells (liver and brain). Among differentially detected lncRNA transcripts, Gm3383 and Gm26870 as a long intergenic non-coding RNA (lincRNA) has very important difference significantly between D and R fractions. Among most significant differentially detected protein-coding transcripts Gm10800, Gm10801, Gm21738, Gm10721, Gm10720, Gm10717, Gm10719, Gm10722, Gm11168, Gm17535, Gm10715 and Lars2-209 are in high levels in D fractions vs. free fractions. Whether the DNA–RNA hybrids identified in sperm are comparable to those identified by immunoprecipitation with S9.6 RNA–DNA-specific antibody followed by sequencing (DRIP-seq) [9,35,36,37,38,39,40,41,42,43] is debatable. None of the functions tentatively attributed to R-loops in transcription control and chromatin structure appears to be relevant in the case of the compacted sperm genome (recruitment of chromatin remodeling factors, activation of transcription by enhanced loading of transcription factors, chromatin decondensation and control of regulatory genes in mouse embryonic stem cells), so that the hybrid structures in sperm DNA are therefore better defined as “inactivated transcription complexes”.

Free transcript profiling in mature sperm represents a subset of RNAs from the round spermatid stage [44]. Sperm chromatin is modified throughout different time points of development [45]. In contrast, transcription activity in the spermatid is gradually stopped and the transcripts remain stable as inactive messenger ribonucleoprotein particles in an inert translational state for several days or longer and eventually translate into elongated spermatids or contribute to the zygote [46,47]. Nuclease footprinting in mouse spermatogenesis revealed that proteins, including the homeobox family members, are probably located within histone-bound chromatin and enriched within genomic regions that undergo transcription in the zygote or 2-cell embryo [48]. Zygotic transcription from the paternal genome pronucleus surpasses that from the maternal genome, supporting the potential role of these sperm carrying regulatory factors in paternal chromatin activation [48,49]. The half-life of each RNA varies from 5 h to at least the time of activation of the embryonic genome, which is the 2-cell stage in the mouse. RNAs that are rapidly degraded upon delivery to the ovum include multiple members of the protamine family that package the genome into a highly condensed transcriptionally inert form. This is likely part of the demolition of the maternal RNAs that occur until the zygotic genome activation [2]. In our study, 1641 transcripts were found at higher levels in D fractions of mature sperm, which indicated that these fragments of transcripts might be more stable in RNA–DNA structures than in free fractions. A limitation of the present study is the number of biological replicates. Further work will be needed with more replicates in order to evaluate the reliability of a differential ratio of D to R transcripts. Furthermore, 257 short RNA fragments of transcription factors are present. Details of these top transcription factors are summarized in Table S3 [50]. Furthermore, the Enrichr analysis (TRANSFAC and JASPAR PWMs) showed 174 short transcripts (Supplementary Materials File S6) in D fractions that are targeted by transcription factor AP-2-alpha (*TFAP2A*), the sequence-specific DNA-binding protein that regulates transcription and binding to the consensus sequence 5′-GCCNNNGGC-3′ and activates genes involved in development [50]. In addition, we observed the presence of many non-coding reads (piRNA and tRNA fragments) associated with the sperm genome in the D fractions.

## 5. Concluding Remark

In the present study, we report that the open region by RNA is maintained along the chromosomes in a silent sperm genome. The essential process that occurs during the first day of the embryo’s life is the replication of the two genomes. Accordingly, insertions of the transcripts are not compatible with the progress of the replication fork, and RNAs are to be removed to complete the first embryonic division. With the evidence for the associations of RNAs in compact sperm DNA, we now confirm that the formation of DNA–RNA hybrids is part of the normal genome life. These results provide an intriguing piece of information on how cells package genomes for transmission. Together, our findings provide a way to identify signals transferred to the oocytes in complex mammalian systems [3,4,5,27,28,29,30,31,33,34]. 

## Supplementary Materials

The following are available online at https://www.mdpi.com/2073-4409/8/10/1196/s1, **Figure S1.** Analysis of next generation sequencing data reveals difference in amount of transcript expression in D and R fractions. MA plot showing log fold change (y-axis) in transcripts: (**A**) for protein-coding; (**B**) for lncRNA between sperm D molecules vs. R fractions compared with the mean of normalized counts (D and R n=4, each samples represents pooled sperm RNA, 2 technical replicates/group). MA plot to visualize RNA-seq data transformed onto the M (log fold change) and A (mean average) scale. Log fold change represents D versus R. **Figure S2.** single lncRNA RNA transcripts in comparison with differential accumulation in sperm D fractions (n = 2 with two replicates). Plotted are the row z-score of log-normalized counts. **Figure S3.** Hierarchical cluster analysis of the pairwise correlation coefficients between the D and R fractions of sperm transcripts. **Figure S4.** (**A**) Pie chart indicating the fraction of exon features (in percent) of D and R sperm fractions; (**B**) Hierarchical clustering showing the correlation between sperm exon features of D and R fractions. **Figure S5.** Hierarchical clustering of non-coding transcripts (piRNA and tRNA-derived fragments). **Figure S6.** IGV visualization of wild-type somatic cells (MEF, D4, D7, iPS, ES) from Cossec et al., 2018 compared to sperm cells (D and R fractions). **Table S1.** Samples Concentration based on A260 measurements before and after RNase H treatment. **Table S2.** Summary of read number. **Table S3**. Primer sequences used for qPCR and relative levels of expression of ten transcripts in mature sperm of *Mus musculus.* **Table S4**. The highest scores related transcription factors. **Table S5**. Details of the known function confirmed transcripts.
