# Supplementary material for: Genome-Wide Distribution of Nascent Transcripts in Sperm DNA, Products of a Late Wave of General Transcription
Source: Cells. 2019 Oct 3;8(10):1196. doi: 10.3390/cells8101196 (PMC6830337; doi:10.3390/cells8101196)
Supplement: Supplementary file 1 [file cells-08-01196-s001.pdf]

Table S1. Samples Concentration based on A260 measurements before and after RNase H treatment.

Unique sample ID	Sample material	Concentration based on A260 measurement (ng/µl)	Concentration based on A260 measurement (ng/µl) after RNase H treatment	Volume (Ml)
**D1**	Mouse Sperm RNA	9	0,001	100
**D3**	Mouse Sperm RNA	6	0,003	100
**R1**	Mouse Sperm RNA	135	129	100
**R3**	Mouse Sperm RNA	50	45	100
**TD1**	Mouse Testes RNA	9	0,005	100
**TD3**	Mouse Testes RNA	15	0,008	100

Table S2. Summary of read number.

**Mapping summary**	**D1_L001**	**D1_L002**	**D3_L001**	**D3_L002**	**R1_L001**	**R1_L002**	**R3_L001**	**R3_L002**
Total reads	65,078,856	66,068,400	69,034,894	69,244,670	77,403,740	77,660,202	87,436,634	87,820,444
Total mapped reads	54,043,408	54,205,377	54,186,775	54,335,469	59,302,998	59,513,909	43,560,556	43,724,836
Map Rate	83.0%	82.0%	78.5%	78.5%	76.6%	76.6%	49.8%	49.8%

Table S3. Primer sequences used for qPCR and relative levels of expression of ten transcripts in mature sperm of *Mus musculus*

**Name**	**Primers Sequence (5'-3')**	**Size (bp)**
Fezf2	Forward: CTGCTCACCCCAAGCTTTTTCTG	152
	Reverse: GTTCAGCTGTCAAGGCCGAG	152
Hmx3	Forward: GGAATCCCCGTTCTCCATCAGG	89
	Reverse: GCAAAGAGCGTTCGTGGGG	89
Hoxb13	Forward: GATCTGCCAGGCTCTGCAG	149
	Reverse: GGCACAGGGTTTCAGGGAG	149
Sox21	Forward: GCTTCTGGAGTGCTCCGG	165
	Reverse: GGCGGGCTGAGGTTTTGAG	165

Nanog	Forward: GCGGTGGCAGAAAAACCAGTG	157
	Reverse: GGTTGGTCCAAGTCTGGCTG	157
UPH	Forward: GGGCATCTGTGGAAAGCAG	165
	Reverse: CCGAGGCCCGCTAACTTAC	165
Lncenc1	Forward: GCAAGGAGTTGACACGATAG	105
	Reverse: CTATGCAGTCTCTGTGCGC	105
Otx-2os1	Forward: GCACATAGGAGGAGAAAGAG	127
	Reverse: CTTGCCTGCTACCAACTGC	127
Platr30	Forward: GGATGCTCGAGATGGTCGG	103
	Reverse: CAGTGTGTGTCGAGCATCC	103
Vmn1r51	Forward: GAGTAAGTGCCCTGAGTAAG	141
	Reverse: CCCTGTGCTGAGGACCTTC	141

Table S4- The highest scores related transcription factors

BSX	(Brain-specific homeobox protein homolog), DNA-binding protein acts as transcriptional activator responsible of normal postnatal growth and nursing
Gsc2	(Homeobox protein goosecoid-2), probably involved in development and autoregulation of transcription
Neurod2	(Neurogenic differentiation factor 2), has a role in the cerebellar and hippocampal granular neurons development
Drgx	(Dorsal root ganglia homeobox protein), essential transcription factor forms correct neuron projections
Lhx5	(LIM/homeobox protein Lhx5), plays an important role in the neuronal differentiation regulation and migration during development of the central nervous system
Barhl2	(BarH-like 2 homeobox protein), regulates neural basic helix-loop-helix genes
Pou6f2	(POU domain, class 6, transcription factor 2), transcription factor probably involved in early differentiation of amacrine and ganglion cells
Fezf1	(Fez family zinc finger protein 1), transcription repressor important for the axonal projection and proper termination of olfactory sensory neurons (OSN). Plays a role in rostro-caudal patterning of the diencephalon and in prethalamic formation. Regulates olfactory bulb development

Table S5- Details of the known function confirmed transcripts

**Transcript**	**Function**
Hoxb13	Encodes homeobox protein Hox-B13 transcription factor, which is part of developmental regulatory system. Specifically generates cells on the anterior-posterior axis [20]
Hmx3	Homeobox protein HMX3 transcription factor. Specifies neuronal cell types that play an important role in inner ear, hypothalamus and hypothalamic/pituitary axis development [20]
Fezf2	Fez family zinc finger protein 2, which is responsible for forebrain embryonic development
Sox21	Plays a role in progressing neurogenesis
Otx2os1	Antisense strands of otx2, which in mice are involved in the development of the midbrain, forebrain and sense organs
Lncenc1	lncRNA that is differentially highly expressed in embryonic stem cells. Probably plays an important role in regeneration of mouse embryonic stem cells. Its depletion is associated with a significant reduction in

	Oct4 and/or Nanog mRNA levels, and it is involved in maintaining the naïve state of embryonic stem cells and may be involved in pluripotency networks [21]
Platr30	Pluripotency associated transcript 30
Uph[1]	Hand2-associated long noncoding RNA, cotranscribed bidirectionally with cardiac transcription factor Hand2, which is an ancestral regulator of heart development [22]

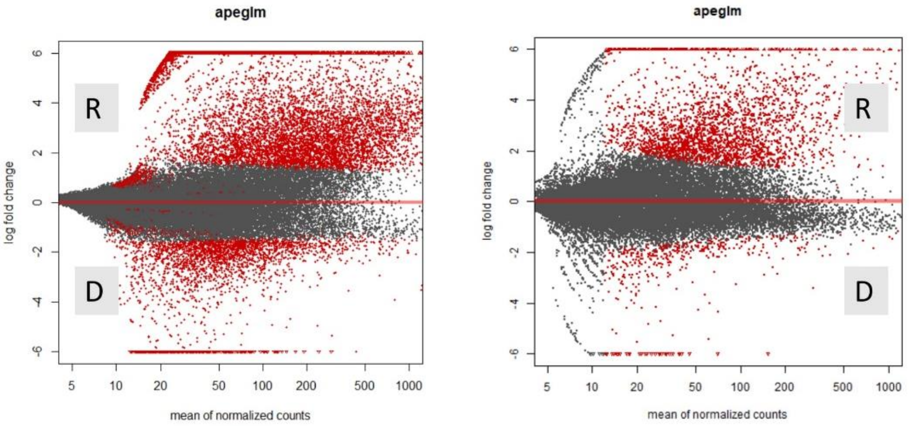

A **B**

Figure S1. Analysis of next generation sequencing data reveals difference in amount of transcript expression in D and R fractions. MA plot showing log fold change (y-axis) in transcripts: (**A**) for protein-coding; (**B**) for lncRNA between sperm D molecules vs. R fractions compared with the mean of normalized counts (D and R n=4, each samples represents pooled sperm RNA, 2 technical replicates/group). MA plot to visualize RNA-seq data transformed onto the M (log fold change) and A (mean average) scale. Log fold change represents D versus R.

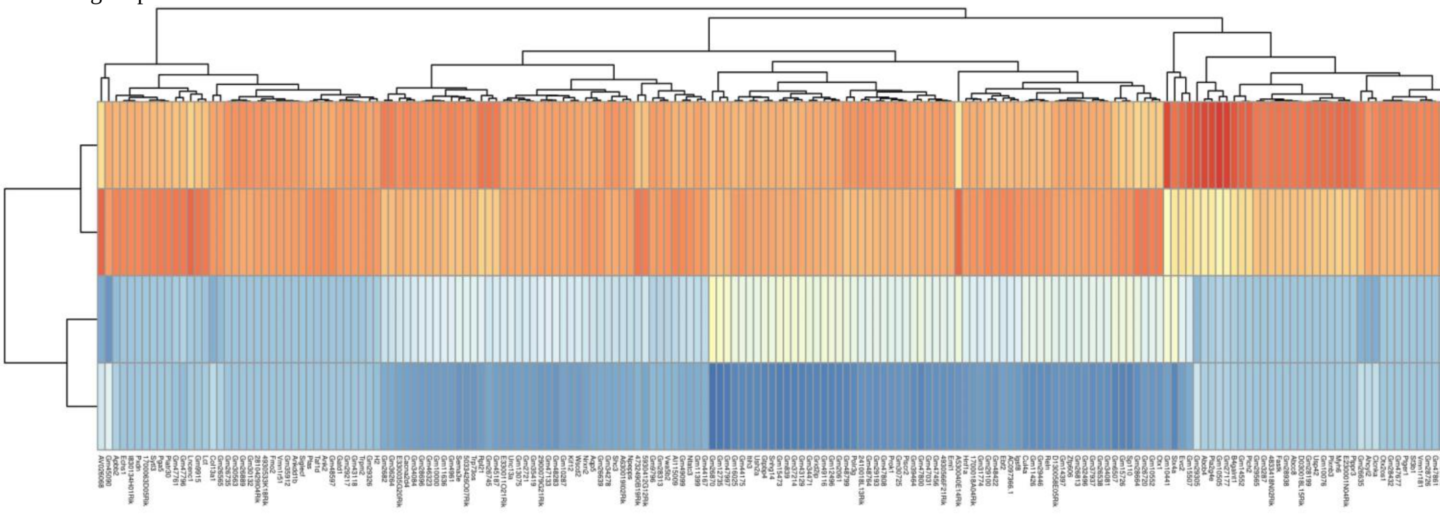

Figure S2. single lncRNA RNA transcripts in comparison with differential accumulation in sperm D fractions (n = 2 with two replicates). Plotted are the row z-score of log-normalized counts.

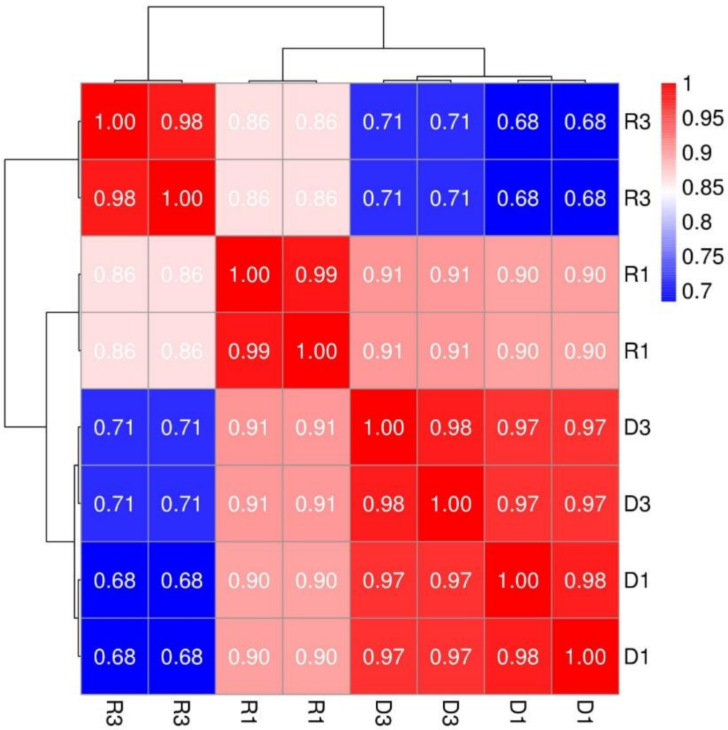

Figure S3. Hierarchical cluster analysis of the pairwise correlation coefficients between the D and R fractions of sperm transcripts.

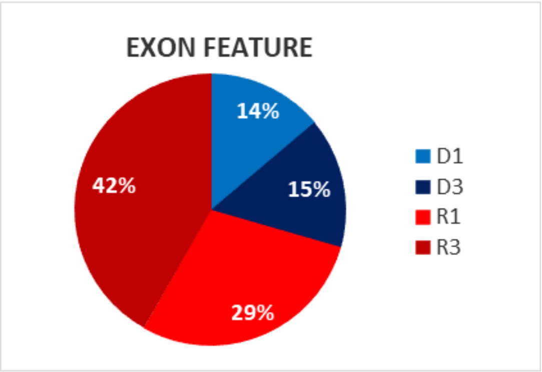

(A)

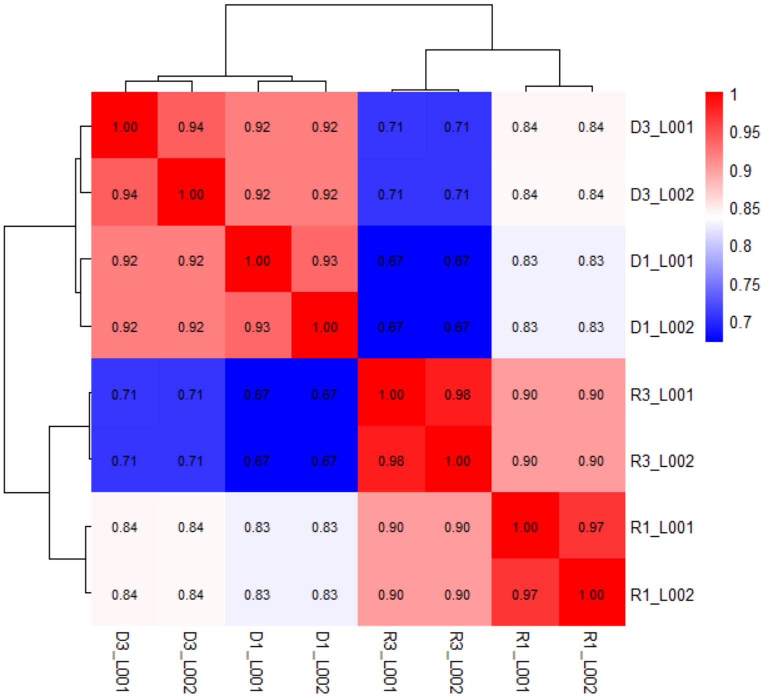

(B)

Figure S4. (**A**) Pie chart indicating the fraction of exon features (in percent) of D and R sperm fractions; (**B**) Hierarchical clustering showing the correlation between sperm exon features of D and R fractions.

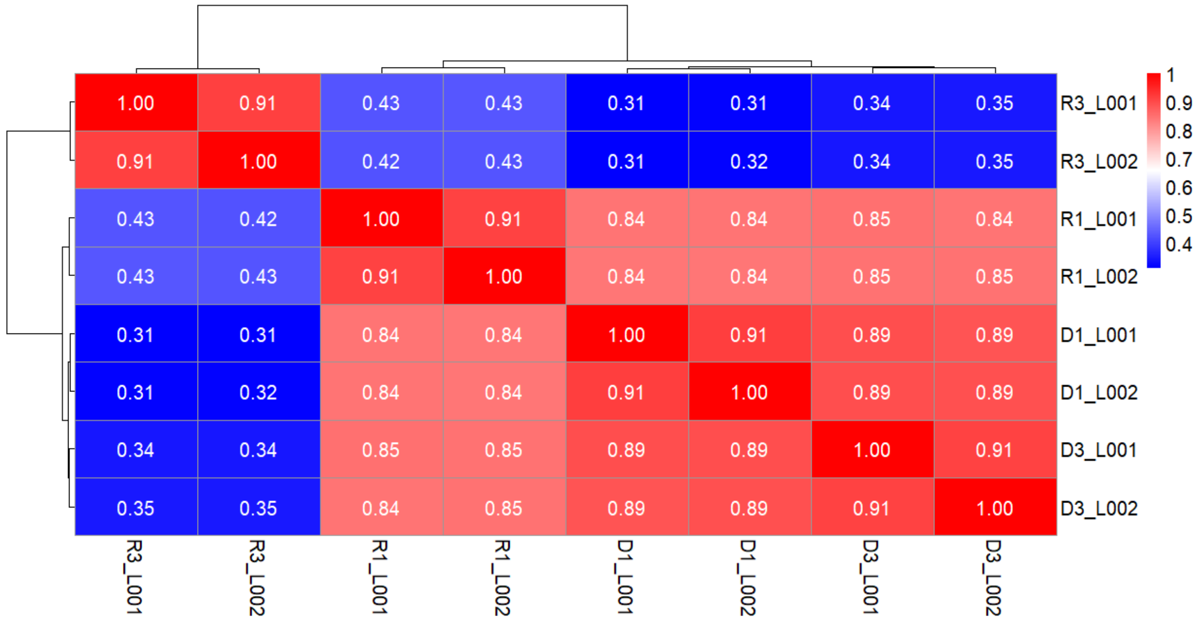

Figure S5. Hierarchical clustering of non-coding transcripts (piRNA and tRNA-derived fragments)

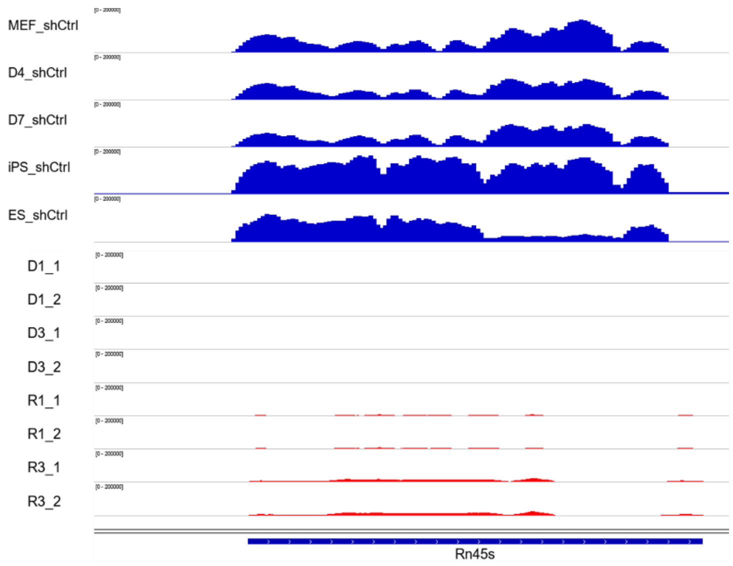

Figure S6. IGV visualization of wild-type somatic cells (MEF, D4, D7, iPS, ES) from Cossec et al., 2018 compared to sperm cells (D and R fractions).
